# How do mini games affect female users of mobile commerce? Improving platform satisfaction through game use intention

**DOI:** 10.3389/fpsyg.2022.973144

**Published:** 2022-09-26

**Authors:** Yi-Ting Huang, Na Yu, Ching-Yi Chen

**Affiliations:** ^1^Department of Interaction Design, National Taipei University of Technology, Taipei, Taiwan; ^2^College of Fine Arts and Design, Wenzhou University, Wenzhou, China; ^3^Department of Commercial Design, Chung Yuan Christian University, Taoyuan, Taiwan

**Keywords:** intention, mini games, mobile commerce, satisfaction, structural equation model, female users

## Abstract

Combining games with mobile commerce applications has been a trend in recent years. Mobile commerce is attracting a large number of people, especially females, to play mini games on the platform. The gamification of mobile commerce may affect users’ platform satisfaction. This study aims to explore the intention of female users to play mobile commerce games and its impact on platform satisfaction. We collected data from females in China who played mobile commerce games and then used a structural equation model to test the various hypotheses we made. The results showed that game use intention fully mediated the impact of performance expectation, hedonic motivation, and social influence on platform satisfaction. Effort expectation had no significant impact on game use intention but had a positive impact on platform satisfaction. Game use intention had a positive impact on platform satisfaction. The frequency of playing games and the amount of money spent on the platform were positively correlated with game use intention. Females over 25 years old preferred to play mobile commerce games. This study provides a theoretical reference and practical enlightenment and makes a specific contribution to the development of mobile commerce platform and the application of gamification. However, this study has limitations in terms of test samples, research methods and research content, and further research on mobile commerce is required in the future.

## Introduction

As a universal form of entertainment, games are a part of people’s daily life. With the rise of the Internet, the channels and ways of playing games have become more diversified to meet the different game needs of people. According to the annual report on mobile apps/games from Seven Wheat Data (2021), the global mobile game market continued to grow rapidly due to the portability and real-time gameplay of mobile devices, with casual games accounting for the largest share of downloads. In 2021, the actual total marketing revenue of the Chinese game market was 296.513 billion yuan, which continues to maintain a fast growth rate. Of this, the actual marketing revenue of the mobile game market was 225.538 billion yuan, which became the main factor driving the overall growth of the game market. The number of game users grew steadily, reaching 666 million, compared with 656 million for mobile games (Game Industry, 2021).

In recent years, the use of games as tools in non-game scenes has become increasingly common. Mobile commerce (m-commerce) has been a mature business phenomenon for a long time, and the marketing community is using gamification to improve customer retention and sales growth. Mini games, such as solving puzzles, keeping virtual pets, and planting virtual trees, have become mainstream games for m-commerce applications. Ant Forest is a green, low-carbon public welfare game launched by Alipay. Virtual energy created by players’ green lifestyle (such as walking, taking buses, and doing things online) is used to raise virtual trees. From 2016 to 2020, the number of participants in Ant Forest grew to more than 550 million, and more than 223 million virtual trees were transformed from Ant Forest and public welfare partners into real trees in desert areas (Ecological and Environmental Research Center, 2021). With 850 million annual active buyers, Pinduoduo was ahead of Alibaba, with 824 million. It took Pinduoduo less than 6 years to achieve the first rank in terms of the number of active buyers of m-commerce applications in China (Net Economics, 2021). With the rapid growth of Pinduoduo, tens of millions of people have been participating in the game Duoduo Orchard for a while. Mini games have played an important role in attracting new users, fission, and promotion. The successful mode of Duoduo Orchard has triggered a wave of mini games launched by Chinese m-commerce applications, such as Jingdong, Taobao, and Vipshop. Nowadays, some international m-commerce platforms also include internal games, such as Wish and Poya.

According to a Baidu game user insight report from Network Game (2021), having free time was the primary factor that led people to play games and compared to males, females preferred leisure games. The mini games of m-commerce belong to the leisure type, with simple game rules and only requiring tens of seconds for a single play. Males play video games more frequently than females, and males spend more time playing games (Griffiths and Lewis, 2011). One reason females spend less time playing games than males is that females have more activities that they are obligated to complete, thus reducing the available leisure time, which reduces the possibility of their freeing up time to play games (Winn and Heeter, 2009). According to previous research, nearly 80% of m-commerce game players are females and needing to spend less time has a positive impact on the game use intention of an m-commerce platform (Yu and Huang, 2022). Therefore, this study selected females as the investigation group to understand the phenomenon of females playing m-commerce games.

In this study, the carriers of the games are m-commerce platforms and such game players themselves are the consumer groups of mobile Internet. Venkatesh et al. (2012) proposed the extended unified theory of acceptance and use of technology model (UTAUT2), and the research object was the consumer group of mobile Internet. However, Venkatesh et al. (2003) proposed the UTAUT based on the comprehensive adoption of eight theories in the last century. Employees who accepted and used new technologies were taken as the research object of UTAUT. Since the research object of UTAUT2 is the consumer group of mobile Internet and the topic of this study is the phenomenon of m-commerce gamification in the 2020s, the research object of UTAUT2 is closer to that of this study, rather than those of the UTAUT model and its previous eight theories. Therefore, this paper chose UTAUT2 as an important theoretical basis. Regarding the literature on game use behavior and willingness, some scholars directly adopted UTAUT2 theory (Ramírez-Correa et al., 2019; Harborth and Pape, 2020) but others modified, deleted, or supplemented research variables based on UTAUT2 to better apply them to specific research situations (Jang and Byon, 2019; Baabdullah, 2020; Sharma et al., 2020; Lin et al., 2022).

There is a lot of literature on satisfaction and behavioral intention. For example, Zhang et al. (2020) pointed out that game satisfaction would affect the willingness of users to keep on playing Ant Forest games. Research by Marinković et al. (2020) showed that customer satisfaction has a significant impact on customer willingness to continue using m-commerce. Satisfaction with m-commerce platforms reflects users’ overall reaction to their experience and their overall evaluation of products and services (Yuan et al., 2016). The platform satisfaction in this study refers to the satisfaction degree after comparing the consumption expectation of the m-commerce platform with the actual experience. Mini games have become an important part of many m-commerce platforms. However, we have not yet found any literature on the impact of willingness to use m-commerce games on platform satisfaction.

Although there have been previous studies on female users, game use intention, and platform satisfaction, no scholars have closely linked these three aspects to discuss the reasons females play m-commerce games and their impact on platform satisfaction. This study believes that it is necessary to carry out detailed research on this comprehensive correlation issue and comprehensively explain the reasons and relations. The main questions this paper addresses are: (1) Why do females play m-commerce games from the perspective of UTAUT2 theory? (2) What is the impact of female willingness to play m-commerce games on platform satisfaction? (3) How can the game use intention and platform satisfaction of female users be further improved through UTAUT2 theory? (4) Are there differences in behavioral intentions among m-commerce game players with different basic demographic characteristics? On the basis of UTAUT2 theory and the concept of satisfaction, this study attempts to establish a research model of females playing m-commerce games.

## Literature review

### Female game use intention

Games have been an integral part of human activities since time immemorial. Playing games is not only a pleasant pastime but also a social activity. Winn and Heeter (2009) argued that for females, playing games must be a satisfying experience to be worth allocating any part of their already scarce free time. In a qualitative analysis of female leisure game players, Griffiths and Lewis (2011) found that game roles and identity are powerful motivators for female players; context and personal experience are the key factors to generate the explanation model of female game motivation. Hartmann and Klimmt (2006) studied female game players and found that females do not like violent content and rigid game characters. Codish and Ravid (2017) found that females have a higher sense of pleasure than males when collecting points and badges. Liao et al. (2020) pointed out that females and high-income gamers are more likely to continue playing online games and the longer they played, the more loyal they became. Compared with females, males are more attracted to different games and play video games more frequently and longer (McLean and Griffiths, 2013). The frequent gaming behavior of online players would gradually approach their game goals, and this motivation to achieve game goals is an effective way to improve players’ willingness to continue playing games (Teng, 2017).

The game use intention in this study refers to the willingness to play m-commerce games. These games are relatively simple and usually do not need mastering difficult game skills or meeting arduous challenges. The games have elements such as points, badges, and leaderboards and provide cash rewards, discounts, and other benefits. Using literature review to analyze relevant papers on the adoption and use of online games, Hamari et al. (2015) found that most variables involve psychological measures related to use, continued use, or behavioral intention. The predictors of using video games were, in order of importance, attitude, flow, satisfaction, and perceived pleasure. Using meta-analysis, Hamari and Keronen (2017) found that attitude, enjoyment, and perceived usefulness were among the variables directly and highly correlated to game intention. Satisfaction, subjective norms, perceived playability, perceived ease of use, and flow were categorized as being moderate correlated. Of course, different types of game players may have different behavioral intentions.

### Construct selection based on UTAUT2

The UTAUT2 model proposed by Venkatesh et al. (2012) includes seven variables, performance expectation, effort expectation, social influence, facilitating conditions, hedonic motivation, price value, and habit, and three moderating variables: gender, age, and experience. The UTAUT2 model involves psychology, sociology, behavior, information system, and other disciplines. It is widely used in information system, e-commerce, marketing, mobile technology, service innovation, and other fields and allows a certain degree of prediction. However, not all factors of the model can fully explain the behavioral intentions of other phenomena. Harborth and Pape (2020) adopted UTAUT2 theory to study Pokémon Go players and found the players’ behavioral intention to be affected by performance expectation, effort expectation, hedonic motivation, convenience, and habit. However, social influence and price value factors could not predict players’ behavioral intention. The research on the combination and expansion of UTAUT2 model and other factors is constantly developing. Algahtani et al. (2021) studied the application of virtual reality technology in medical centers from the perspective of UTUAT2 theory but deleted the price value variable of UTUAT2 theory and added two variables: individual innovation and satisfaction.

The gamification of m-commerce in this study aims to motivate users to buy and pay. Playing games may help players purchase goods on the platform at a reduced cost or get additional payment rewards. Therefore, this study integrated the price value dimension of UTAUT2 into the performance expectation dimension and did not take price value as a separate dimension in the empirical study. M-commerce games have social attributes that can enhance the community communication of products through sharing and enhance user participation through competition and cooperation. Therefore, this study taken the social influence dimension as an important research variable. Providing an interesting and enjoyable environment can help improve users’ understanding of technology acceptance (Venkatesh et al., 2012). M-commerce games provide entertainment and relaxation, so the hedonic motivation variable of UTAUT2 is used in this study.

In the early stage of m-commerce, facilitating condition is an important factor affecting people’s use intention. However, nowadays, people have relatively abundant equipment and resources to use mobile applications. For example, studies by Ramírez-Correa et al. (2019) and Oh and Yoon (2014) have shown no significant relationship between facilitating conditions and behavioral intention to play online games. Therefore, the facilitating condition variable of UTAUT2 was not included in this research model. In this study, the subjects were people who frequently played games on the m-commerce platform and most of them had formed the habit of completing the tasks assigned by games. This behavior encouraged players to play the game repeatedly and kept them engaged in the game for a long time so they could gradually achieve their goals (Teng, 2017). Therefore, we did not include the habit variable of UTAUT2 into this research model.

To sum up, UTAUT2 theory was selected as an important reference in this study. On this basis, combined with the specific situation of gamification of m-commerce, research variables slightly different from previous studies were proposed: performance expectation (including price value), effort expectation, social influence, and hedonic motivation.

### Development of hypotheses

As a strong predictor of intention, performance expectation helps to identify users’ behavioral intentions (Martins et al., 2014; Wong et al., 2015; Aydin, 2018; Tan and Ooi, 2018). Relevant studies have confirmed the importance of performance expectation for players’ willingness to use games in the online game environment (Marham and Saputra, 2019; Harborth and Pape, 2020). The price value, which was a part of the performance expectation dimension in this study, was considered to be one of the key factors affecting the intention to use e-sports games (Jang and Byon, 2019). In a study on a self-service lending and returning system for libraries, it was found that performance expectation had a positive influence on continuous use intention and satisfaction (Wu and Wu, 2018). Therefore, the following hypotheses were derived in this study:

*H1a*: Performance expectancy is positively related to game use intention.

*H1b*: Performance expectancy is positively related to platform satisfaction.

Many scholars believe that hedonic motivation is a strong trigger in the context of online games and has a significant impact on players’ willingness to continue (Wang and Goh, 2017; Ramírez-Correa et al., 2019; Baabdullah, 2020). In a study on the behavior and motivation of Pokémon GO players, it was found that enjoyment, community involvement, network externalities, and collection needs significantly affected players’ willingness to continue using (Ghazali et al., 2018). Zhou (2011) pointed out that perceived enjoyment has a strong impact on user satisfaction, thus affecting the continuous use of the mobile Internet. Satisfaction had a positive influence on users’ willingness to keep on using Ant Forest game. Environmental concern, perceived enjoyment, and game interaction had a positive impact on satisfaction (Zhang et al., 2020). Therefore, the following hypotheses were derived:

*H2a*: Hedonic motivation is positively related to game use intention.

*H2b*: Hedonic motivation is positively related to platform satisfaction.

Social interaction can form flow, fun, and social influence, which can generate a positive attitude among players toward the game so as to encourage them to continue to use the game (Choi and Kim, 2004; Alha et al., 2019; Lee H. et al., 2021). M-commerce games usually contain strong social functions. Online gamers often need to rely on each other to overcome game challenges, thus establishing interdependent relationships (Teng et al., 2012). Social factors influence the continuous use of the mobile Internet, and social motivation is positively correlated with the use of live video game platforms (Cabeza-Ramírez et al., 2020). On the basis of the perspective of UTAUT, Marinković et al. (2020) found that social influence is positively correlated with satisfaction with m-commerce. Therefore, the following hypotheses were proposed:

*H3a*: Social influence is positively related to game use intention.

*H3b*: Social influence is positively related to platform satisfaction.

Relevant studies have confirmed that effort expectation is one of the important determinants of playing online games on mobile phones and may positively affect behavioral willingness (Chen and Kuan, 2012; Oh and Yoon, 2014; Jang and Byon, 2019). Providing a pleasant experience is the focus of hedonic games, while perceived ease of use and perceived usefulness are the main determinants that encourage people to accept utilitarian games (Wang and Goh, 2017). The game carrier of this study is an m-commerce platform, and mini games are actually utilitarian, which aims to motivate users to buy and pay on the platform. Research by Ramayah and Ignatius (2005) showed that when predicting the willingness of potential e-shoppers, it was essential to identify the ease of use of technology and the satisfaction of shoppers with the online shopping experience. Therefore, the following hypotheses were proposed:

*H4a*: Effort expectancy is positively related to game use intention.

*H4b*: Effort expectancy is positively related to platform satisfaction.

Previous studies have shown that satisfaction has a significant impact on the willingness of a player to continue using some platforms, such as mobile shopping apps, metaverse platform, online social networks, and book self-service systems (Natarajan et al., 2017; Wu and Wu, 2018; Lee and Kim, 2022). Satisfaction with the mobile shopping application environment is the sum of emotional responses to mobile shopping application activities triggered by various factors, such as information, system, and service quality (Agrebi and Jallais, 2015). In a study on m-commerce, Lu (2014) found that the ultimate success of m-commerce, in terms of ensuring continuous use, depends on improving user satisfaction. Kumar and Acharjya (2017) pointed out that game satisfaction and game system quality significantly affect the behavioral intention to adopt online games. Therefore, the following hypothesis was proposed:

*H5*: Game use intention is positively related to platform satisfaction.

## Materials and methods

### Sample and instrument development

The subjects of this study were females who played m-commerce games, so the title of the questionnaire emphasized the word “female” and the first question was designed as a gender screening question. If the subject chose the male option, the system automatically terminated the questionnaire. In addition, we found in the pre-test that females who rarely played m-commerce games tended to score lower on the game use intention option. Therefore, in the formal research stage, we set a question regarding how often they had played m-commerce games in the past 6 months, providing five options: never played, rarely played, 3–5 times a month, 2–4 times a week, and every day. If the respondent chose the option never played or rarely played, the questionnaire would stop automatically. The pre-test questionnaire in this study contained items from the habit dimension of UTAUT2 theory. In the formal survey stage, only females who played m-commerce games frequently were accepted, so there was no question related to the research variable “habit” in the formal questionnaire. By default, all the subjects had the habit of playing m-commerce games.

On the basis of the classification framework of age, education level, frequency of use, and residence, this study paid to promote the questionnaire content on a nationwide questionnaire distribution platform in April 2022. This part was probability sampling. In addition, respondents could forward the questionnaire to their friends for a small reward. This part was snowball sampling. After removing the questionnaires where the participants had taken too short a time to fill out the forms, illogical questionnaires, and the questionnaires with all same-scale items, we were left with 609 valid questionnaires. The statistical results showed that females from 28 provincial-level administrative regions in China participated in the survey, which ensured that the research sample was sufficiently representative.

In the measurement of UTAUT2, game use intention, and platform satisfaction, we referred to the scale items of international published academic literature and made appropriate modifications based on the research topics so as to adapt the items to the purpose of this study. A five-point Likert scale was used in the questionnaire, in which 5 indicated absolute agreement and 1 indicated absolute disagreement. The specific contents of the scale are shown in Table 1. The research plan, the consent form, and the questionnaire used in this study were reviewed by the Research Ethics Committee. The participants completed the informed consent forms in this study.

**Table 1:** Summary of factor analysis.

Construct	Items	Std.	SMC	AVE	CR
Performance expectancy (M = 4.087, SD = 0.874)	It is useful to play m-commerce games (to entertain, save money, promote social interaction, protect the environment, etc.).	0.730	0.533	0.572	0.870
	The m-commerce games I play bring the desired value.	0.802	0.643		
	M-commerce games allow me to get good shopping discounts.	0.767	0.589		
	M-commerce games give me a higher chance to shop more cheaply.	0.735	0.541		
	M-commerce games allow me to acquire useful information to shop more wisely.	0.744	0.554		
Effort expectancy (M = 4.409, SD = 0.668)	How to play m-commerce games is easy.	0.761	0.578	0.525	0.816
	I can easily master how to play m-commerce games.	0.714	0.510		
	I think the gameplay of m-commerce games is simple.	0.721	0.519		
	Playing m-commerce games is easy.	0.702	0.493		
Social influence (M = 3.964, SD = 0.966)	Many people around me are playing m-commerce games.	0.756	0.571	0.571	0.780
	My friends and family would talk to me about m-commerce games.	0.778	0.605		
	I may play m-commerce games because of the influence of others.	0.732	0.536		
Hedonic motivation (M = 4.180, SD = 0.825)	M-commerce games bring me happiness.	0.792	0.627	0.620	0.891
	M-commerce games are enjoyable.	0.784	0.615		
	M-commerce games can be relaxing.	0.772	0.595		
	M-commerce games are fun.	0.804	0.646		
	M-commerce games are interesting.	0.785	0.616		
Game use intention (M = 4.119, SD = 0.861)	I am interested in playing m-commerce games.	0.806	0.650	0.651	0.882
	I like playing m-commerce games.	0.822	0.622		
	I may keep on playing m-commerce games.	0.788	0.675		
	I tend to make positive comments about m-commerce games.	0.810	0.656		
Platform satisfaction (M = 4.266, SD = 0.623)	I am satisfied with the overall experience of the m-commerce platform.	0.715	0.511	0.530	0.819
	My overall feeling when using the m-commerce platform is pleasant.	0.718	0.515		
	My overall feeling when using the m-commerce platform is satisfactory.	0.772	0.596		
	The experience of the m-commerce platform is what I want.	0.706	0.498		

Data were analyzed using Amos 22 Graphics and SPSS Statistics 26 software. In this study, there were 6 latent variables and 25 observed items in the structural equation model. If the sample number suggested by Hair et al. (2010) was 15 to 20 times the number of observed variables, the 609 valid samples in this study were considered to have met the sample size requirements, so it is appropriate to adopt structural equation model. Maximum likelihood was chosen as the estimation method. The structural equation modeling method was used to verify the proposed hypothesis. The test procedure proposed by Baron and Kenny (1986) was used to test the intermediate variables.

### Descriptive statistics

In this study, it was found that the types and proportions of m-commerce platforms that respondents usually used were Taobao (96.39%), Alipay (85.22%), Pinduoduo (79.64%), and others (9.52%). In terms of the multiple-choice questions on which m-commerce games were played recently, the types and proportions of platforms the respondents selected were Taobao (87.03%), Alipay (84.40%), Pinduoduo (64.04%), and others (7.09%). As to which type of m-commerce games they had played, the types and proportions of the games the respondents selected were Ant Forest (87.52%), growing fruits (84.73%), solving puzzles (68.47%), raising pets (53.69%), and others (4.96%). The above data indicate that the game types and m-commerce platforms selected in this study were sufficiently representative. In addition, 92.61% of the respondents indicated that they had played independent applications that were similar to m-commerce games before.

Table 2 provides descriptive statistics regarding the respondents. In this sample, 69.95% of the population was older than or equal to 25 years old. By level of education, 81.77% of the respondents were undergraduates or from junior college. Most respondents played games on the m-commerce platform every day or 2–4 times a week. Most of the subjects spent more than 201 RMB per month on the platform.

**Table 2:** Descriptive statistics of the participants.

Measure	Rank	Frequency	Percentage
Age	<25 years	183	30.05
	≥25 years	426	69.95
Educational level	High school or below	58	9.52
	Undergraduate or junior college	498	81.77
	Postgraduate or above	53	8.70
Frequency of playing m-commerce games	Every day	261	42.86
	Two–four times a week	277	45.48
	Three–five times a month	71	11.66
Average monthly platform expenditure	≤200 RMB	108	17.73
	>201 RMB and ≤500 RMB	276	45.32
	≥501 RMB	225	36.95

## Results

### Reliability and validity analysis

The reliability and validity of the research model should be analyzed before the structural model. As the questionnaire scale of this study was designed for the research theme and object characteristics based on the relevant literature, exploratory factor analysis was required for the data before confirmatory factor analysis. In this study, principal component extraction was used to extract six factors, which accounted for 68.813% of the total variance. The Kaiser–Meyer–Olkin value of this study was 0.940, and Bartlett’s sphericity test was significant (*p* < 0.001), indicating that the data used were suitable for factor analysis (Kaiser, 1974). Cronbach’s alpha values of each dimension ranged from 0.796 to 0.892, all being greater than 0.7, indicating that there was a high degree of internal consistency among the items of the dimensions and confirming the reliability of the structure (Nunnally, 1994).

Analysis was carried out using Amos software, and the reliability and validity values of each dimension and item are shown in Table 1. The composite reliability (CR) of all dimensions in this study ranged from 0.780 to 0.891, all greater than 0.7, showing high component reliability. Therefore, the observed variables were sufficient to reflect the potential variables, which indicated that the research model had good internal consistency (Fornell and Larcker, 1981). The standardized factor loading (Std.) of all the items ranged from 0.702 to 0.822, all values greater than 0.7, showing the ideal state (Hair et al., 2010).

Discriminant validity analysis was used to verify whether there is a high degree of correlation between different dimensions. When the average variance extraction (AVE) square root of a dimension was greater than the correlation coefficient between this dimension and other dimensions, it indicates that there was discriminant validity between each dimension (Fornell and Larcker, 1981). It can be seen from Table 3 that this model has good discriminant validity. According to the above results, the reliability and validity of all variables in this study were at an acceptable level, so this model has enough reliability and validity.

**Table 3:** Correlations of variables.

Variables	SI	EE	PE	HM	GUI	PS
SI	**0.756**					
EE	0.317	**0.725**				
PE	0.552	0.326	**0.756**			
HM	0.663	0.465	0.710	**0.787**		
GUI	0.632	0.360	0.713	0.773	**0.807**	
PS	0.377	0.517	0.413	0.489	0.544	**0.728**

Square roots of the AVE values are reported in bold on the diagonal.

### Structural model analysis

The overall model fit analysis results of this study are as follows. CMIN = 472.957, CMIN/df = 1.792, GFI = 0.943, AGFI = 0.930, SRMR = 0.0360, RMSEA = 0.036, NFI = 0.942, IFI = 0.974, CFI = 0.974, RFI = 0.935, and PNFI = 0.829. It can be seen that the indicators of absolute fit measures, incremental fit measures, and parsimonious fit measures in this study all performed well, meeting the requirements of relevant academic research (Hair et al., 2010).

**Figure 1:**
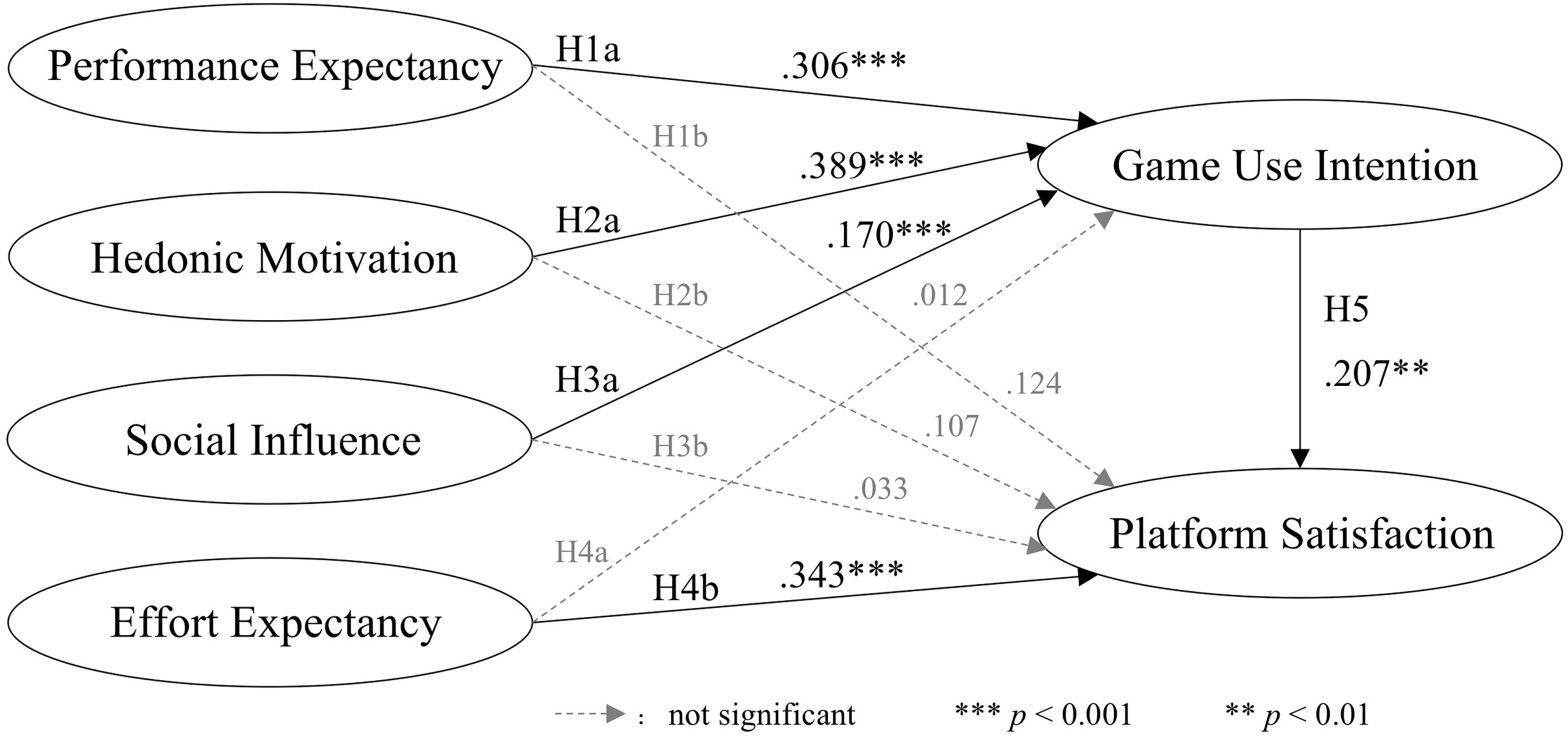
Empirical test results.

The structural equation model was used to examine the research conceptual model, and Figure 1 shows the research result diagram. The results show that performance expectancy (β = 0.306, t = 5.979, *p* < 0.001), hedonic motivation (β = 0.389, t = 6.220, *p* < 0.001), and social influence (β = 0.170, t = 3.469, *p* < 0.001) all positively affected the game use intention. Therefore, the results supported H1a, H2a, and H3a. Effort expectancy (β = 0.343, t = 6.567, *p* < 0.001) positively affected platform satisfaction, so the result supported H4b. However, performance expectation (β = 0.124, t = 1.824, *p* > 0.05), hedonic motivation (β = 0.107, t = 1.280, *p* > 0.05), and social influence (β = 0.033, t = 0.527, *p* > 0.05) had no significant influence on platform satisfaction. Therefore, H1b, H2b, and H3b were rejected. Effort expectancy had no significant impact on game use intention, so H4a was rejected. In addition, game use intention was positively related to platform satisfaction (β = 0.207, t = 2.608, *p* < 0.01), so the results supported H5. The hypothesis test results are shown in Table 4.

**Table 4:** Path analysis.

Path	β	t	*p*	Result
performance expectancy → game use intention	0.306	5.979	***	Support H1a
hedonic motivation → game use intention	0.389	6.220	***	Support H2a
social influence → game use intention	0.170	3.469	***	Support H3a
effort expectancy → game use intention	0.012	0.247	0.805	Reject H4a
performance expectancy → platform satisfaction	0.124	1.824	0.068	Reject H1b
hedonic motivation → platform satisfaction	0.107	1.280	0.201	Reject H2b
social influence → platform satisfaction	0.033	0.527	0.598	Reject H3b
effort expectancy → platform satisfaction	0.343	6.567	***	Support H4b
game use intention → platform satisfaction	0.207	2.608	**	Support H5

**p < 0.01, ***p < 0.001.

We tested the mediating effect of the research model. The results showed that game use intention acted as a full mediation in the impact of performance expectation, hedonic motivation, and social influence on platform satisfaction. Performance expectancy (β = 0.124, t = 3.440, *p* < 0.001), hedonic motivation (β = 0.183, t = 3.765, *p* < 0.001), and social influence (β = 0.069, t = 2.294, *p* < 0.001) had a significant indirect impact on platform satisfaction.

The game use intention of females over 25 years old (M = 4.163) was significantly higher than that of females under 25 years old (M = 4.018). There were significant differences in the willingness of users to play games on m-commerce platforms daily (M = 4.380), 2–4 times per week (M = 4.005), and 3–5 times per month (M = 3.602). The game use intention of users who spent more than 501 yuan (M = 4.273) on the mobile commerce platform every month was significantly higher than that of users who spent less than 200 yuan (M = 4.028) and 201–500 yuan (M = 4.030). However, regarding game use intention, there was no significant difference among game players with different education levels. In addition, the effect of game use intention on platform satisfaction was not moderated by age, game frequency and educational level. However, the effect of game use intention on platform satisfaction was moderated by monthly platform expenditure (the higher consumption group was more significant).

## Discussion and conclusion

### Theoretical and managerial implications

In the context of m-commerce incorporating mini games, this study established a research model and put forward hypotheses from the perspective of UTAUT2 theory. On the basis of questionnaire data, the behavioral intention of females using m-commerce games and its influence on platform satisfaction were investigated. This study confirmed that game use intention had a full mediating effect between performance expectation, hedonic motivation, social influence of the self-variable and platform satisfaction of the dependent variable. Effort expectation had no significant impact on game use intention but had a positive and significant impact on platform satisfaction. According to the relevant demographic results, further explained the behavior intention of female m-commerce game players. The results of this study are expected to supplement and expand the research in related fields and contribute to the development of m-commerce and the application of games.

In view of the game characteristics and users of m-commerce, this study modified the research variables of the UTAUT2 model. In the revised model, we incorporated the variable of price value into performance expectation, deleted the two variables of convenience facilitation and habit, and retained the three variables of effort expectation, hedonic motivation, and social influence. In this study, the modified UTAUT2 model was used as the self-variable, the game use intention as the intermediary variable, and the platform satisfaction as the dependent variable. The collected data had good model fitness and explanatory power through empirical analysis. It extends the application of UTAUT2 to user-related research and is an important contribution of this study.

M-commerce games are utilitarian products. The games involve browsing the advertising page, purchasing goods, signing in, donating the number of walks, and so on. The player has to solve the challenges in the game by completing tasks. Appropriate incentives can further motivate players to complete tasks within the time limit. Games help to visually explain complex tasks or features, engaging participants through competition, teamwork, curiosity, and problem solving (Boinodiris, 2012). Providing the value users expect as the attraction point can improve their willingness to continue playing m-commerce games and enhance platform satisfaction. Combined with the characteristics of the game industry, cross-border integrated development will become a new way to improve the profitability of the industry, to achieve a win–win or even a multi-win situation.

The reason females play m-commerce games is not only to gain some utilitarian value but also to enjoy the mini games themselves, for example, *via* a sense of pleasure and achievement. According to the classification of Lazzaro (2009) game fun, in general, gamers on m-commerce platforms experience easy fun in the early stages of game play and hard fun in the middle and late stages. It is also possible to have fun interacting with others and enjoy the fun of receiving real rewards, such as free fruit, after winning the game. Female consumers tend to enjoy the elements of gamification more than males (Jakobs, 2016). Providing an interesting game environment can also indirectly increase platform satisfaction among females in m-commerce.

M-commerce mini games require little or no attention and can be played while multitasking. Winn and Heeter (2009) argued that females are more likely than males to engage in social or other beneficial activities in their spare time and games that allowed them to socialize with friends or family while multitasking may appeal to females. Mini games of m-commerce spread rapidly in the form of social fission, which can effectively attract people to play games on the platform in the short term. Point accumulation, badge upgrade, and leaderboard promote people’s social interaction (Xi and Hamari, 2019). Social influence encourages players to continue to participate in activities, which may virtually have a positive influence on platform satisfaction.

The study results show that the performance expectation, hedonic motivation and social influence have no direct and significant impact on platform satisfaction of m-commerce, but game use intention can significantly affect platform satisfaction. This finding is not completely the same as the research results of Kalinić et al. (2019). In the previous research, Kalinić et al. (2019) found that performance expectation directly and significantly affects the users’ satisfaction of m-commerce, while hedonic motivation and social influence have no direct and significant impact on the users’ satisfaction of m-commerce. This is because the m-commerce with gamification attributes is highlighted in this study, rather than the m-commerce without gamification attributes in the study of Kalinić et al. (2019). There are differences between the results of the two studies. Similarly, Tandon et al. (2018) and Lee W. et al. (2021) indicated that the same self-variable may not have a consistent effect on satisfaction in different contexts.

The empirical results of this study indicate that effort expectation had no significant impact on game use intention of the m-commerce platform, which was consistent with the research results of Ramírez-Correa et al. (2019) and Wang et al. (2020). However, another finding was that effort expectations positively affect platform satisfaction. Research by Yu and Huang (2022) showed that the game use intention of m-commerce had a positive and significant influence on the platform purchase intention. Satisfaction is regarded as an important factor in the online shopping research model because it may affect participants’ motivation to stay on the platform and is considered as the premise of repurchase (Hsu et al., 2015). M-commerce games are designed to guide and motivate players to shop and pay on the platform and not just play games. The simple attributes of the games ensure that users do not shy away from what they want to achieve. However, it is difficult for easy games to continuously have a strong appeal for players. This explains why effort expectation cannot directly increase the willingness of female m-commerce players to use games but can increase their platform satisfaction.

Studies on game use intention and satisfaction simultaneously usually take independent game apps as objects and explore the influence of game satisfaction on game use intention (Kumar and Acharjya, 2017; Hsiao et al., 2019; Zhang et al., 2020). However, this study illustrates that game use intention has a positive and significant impact on the satisfaction with m-commerce platform, which fills the research gap in related fields. The results of this study show that only monthly platform expenditure has a significant moderating effect on the effect of game use intention on platform satisfaction, while age, game frequency and educational level do not have such effect. Previous literature has also used UTAUT2 theory to discuss game use intention and satisfaction (Oh and Yoon, 2014; Ramírez-Correa et al., 2019; Marinković et al., 2020; Barbosa et al., 2021). However, there are few studies on females playing m-commerce games and m-commerce platform satisfaction using game use intention as a mediating variable, and there are few studies on the moderating effect between game use intention and platform satisfaction from the perspective of demographics. This provides novel insights into mobile commerce.

The research results confirmed that the frequency of playing games is directly proportional to game use intention. This is consistent with previous research showing that game use intention of m-commerce has a positive influence on platform purchase intention (Yu and Huang, 2022). The results of this study indicated that female gamers over the age of 25 years are significantly more willing to use games than female gamers under the age of 25. This is the same as the result of research by Liao et al. (2020), which showed that high-income game players prefer to continue playing online games. However, there is no significant difference in game use intention among subjects with different levels of education, which is consistent with the research results of Zhang et al. (2020) on Ant Forest games.

This study makes some academic theoretical contributions, reveals the reasons why females play m-commerce games, and confirms that game use intention positively affects the satisfaction with m-commerce platforms, thus filling the gaps in academic research in related fields. For m-commerce platforms incorporating game attributes, a deeper understanding of the potential causes behind the behavior of female gamers will help more effectively predict and guide user behavior and provide reference for management decisions to improve the satisfaction with m-commerce platforms. The research results can not only provide a reference basis for China’s m-commerce but also be potentially applied to the international market. This may help enterprises seek new competitive value by using game methods, so as to obtain more long-term competitive advantages.

### Conclusion, limitations, and suggestions for future research

In this era of vigorous m-commerce development, this study constructed a research model from the perspective of the revised UTAUT2 theory to explain the reasons females play m-commerce games and its impact on platform satisfaction. The hypothesis relationship was verified by path analysis and was credible and persuasive to an extent. The results showed that performance expectation, hedonic motivation, and social influence have no direct effect on platform satisfaction but can indirectly affect platform satisfaction through game use intention. Effort expectation had no direct and significant impact on game use intention but had a positive and significant impact on platform satisfaction. This study also further explored game use intention and platform satisfaction in terms of age, playing frequency, education level, and monthly spending on the platform. The overall model contributes to explaining the games and satisfaction with the m-commerce platforms.

However, this study has the following limitations: (1) From the empirical aspect, the test sample of this study was limited to some female users who played games on the Taobao, Pinduoduo, and Alipay m-commerce platforms. Since mainstream m-commerce platforms and built-in mini games in other countries or regions may be different, as would be people’s living habits and ways of thinking and the factors influencing their behavior, whether the results of this study can be replicated among people in other countries or regions still needs to be verified by subsequent empirical studies. It is necessary to expand the research scope to different m-commerce platforms or regions in the future. (2) We collected the questionnaire samples of this study within a few days and analyzed the data using SEM. In the future, other methods, such as longitudinal study, can be adopted to track the follow-up dynamics of target users through irregular interviews or questionnaires so as to gradually overcome the limitations of this study. (3) The categories of games in this study were Ant Forest, growing fruits, raising pets, and solving puzzles, but there was no detailed discussion and comparison on the use intention and users’ reasons behind playing each category of games. Future research can further study the behavior and willingness of players in terms of different m-commerce games and put forward specific and feasible design suggestions for game developers.

## Data availability statement

The original contributions presented in the study are included in the article/Supplementary material, further inquiries can be directed to the corresponding author.

## Ethics statement

The studies involving human participants were reviewed and approved by Research Ethics Committee of Quanzhou Normal University. Written informed consent to participate in this study was provided by the participants’ legal guardian/next of kin.

## Author contributions

Y-TH, NY, and C-YC contributed to conception and design of the study. NY organized the database, performed the statistical analysis, and wrote the first draft of the manuscript. Y-TH and NY wrote sections of the manuscript. Y-TH edited the manuscript. All authors contributed to the article and approved the submitted version.

## Conflict of interest

The authors declare that the research was conducted in the absence of any commercial or financial relationships that could be construed as a potential conflict of interest.

## Publisher’s note

All claims expressed in this article are solely those of the authors and do not necessarily represent those of their affiliated organizations, or those of the publisher, the editors and the reviewers. Any product that may be evaluated in this article, or claim that may be made by its manufacturer, is not guaranteed or endorsed by the publisher.
